# Human pluripotent stem cell-derived macrophages and macrophage-derived exosomes: therapeutic potential in pulmonary fibrosis

**DOI:** 10.1186/s13287-022-03136-z

**Published:** 2022-09-02

**Authors:** Roya Rasaei, Apoorvi Tyagi, Shima Rasaei, Seung-Joon Lee, Se-Ran Yang, Kye-Seong Kim, Suresh Ramakrishna, Seok-Ho Hong

**Affiliations:** 1 Department of Internal Medicine, School of Medicine, Kangwon National University, 1 Kangwondaehakgil, Chuncheon, Gangwon-do, 24431 South Korea; 2 Graduate School of Biomedical Science and Engineering, Hanyang University, Seoul, 04763 South Korea; 3 Department of Cellular and Molecular Science, Falavarjan Branch, Islamic Azad University, Falavarjan, Iran; 4 Department of Thoracic and Cardiology, School of Medicine, Kangwon National University, Chuncheon, 24341 South Korea; 5 Institute of Medical Science, Kangwon National University, Chuncheon, 24341 South Korea; 6 KW-Bio Co., Ltd, Wonju, South Korea

**Keywords:** Macrophages, IPF, hPSC, Exosomes, Respiratory disease

## Abstract

Pulmonary fibrosis (PF) is a fatal chronic disease characterized by accumulation of extracellular matrix and thickening of the alveolar wall, ultimately leading to respiratory failure. PF is thought to be initiated by the dysfunction and aberrant activation of a variety of cell types in the lung. In particular, several studies have demonstrated that macrophages play a pivotal role in the development and progression of PF through secretion of inflammatory cytokines, growth factors, and chemokines, suggesting that they could be an alternative therapeutic source as well as therapeutic target for PF. In this review, we describe the characteristics, functions, and origins of subsets of macrophages involved in PF and summarize current data on the generation and therapeutic application of macrophages derived from pluripotent stem cells for the treatment of fibrotic diseases. Additionally, we discuss the use of macrophage-derived exosomes to repair fibrotic lung tissue.

## Introduction

Macrophages are immune cells that originate from progenitors in the bone marrow (BM) that circulate in the peripheral blood and migrate into different tissues [1–4]. Macrophages are the foremost controllers of both innate and acquired immunity and are activated by different endogenous and exogenous signals to mediate immune homeostasis [5]. Macrophages are major mediators of inflammation, tissue repair, and immune function due to their ability to secrete an array of soluble cytokines, chemokines, and growth factors. Specialized macrophages in different tissues are referred to by different names: liver macrophages that promote tissue remodeling and immune responses are called Kupffer cells, macrophages that maintain the immunity of the brain by eliminating dead neurons are referred to as neuronal macrophages or microglia, macrophages that eliminate dysfunctional or old red blood cells (RBCs) are called splenic macrophages, while macrophages that phagocytose dead cells or bacteria in lung tissue are referred to as lung macrophages [6].

Macrophages are abundant in the lung microenvironment and comprise a heterogeneous population of cells with diverse functions and phenotypic plasticity dependent on the inflammatory signals they encounter in the lung microenvironment. Lung macrophages are important sentinels that are critical to pulmonary host defenses. Two macrophage populations reside within lungs: alveolar macrophages (AMs) and interstitial macrophages (IMs), which differ in the expression of surface markers as well as their localization and functional phenotype [7, 8]. AMs are primary effector cells that possess both pro-inflammatory and anti-inflammatory properties and express low levels of CD11b and high CD11c levels and colonize the airway space in lungs. By contrast, high CD11b- and CD11c-expressing IMs reside in the lung parenchyma and maintaining immune homeostasis in the respiratory tract [9]. Inflammatory responses of macrophages are associated with the development of acute and chronic pulmonary pathologies including idiopathic pulmonary fibrosis (IPF) [10, 11]. In this review, we describe the characteristics, functions, and origins of subsets of macrophages and discuss the importance and regulation of macrophage polarization in the development of IPF. Additionally, we summarize the current state of knowledge regarding the therapeutic use of pluripotent stem cell-derived macrophages to treat fibrotic diseases and macrophage-derived exosomes to repair fibrotic lung tissue.

## Role of macrophages in lung fibrosis

### IPF

IPF is the most common lung disease and is characterized by the progressive deposition of collagen and extracellular matrix, resulting in damaged and scarred lung tissue. The scarring associated with IPF can lead to impaired gas exchange, breathlessness, decreased static lung compliance, and respiratory failure [12–14]. A number of genetic and non-genetic factors contribute to the development of IPF [15]. Non-genetic factors such as cigarette smoking, dust exposure, and infection are believed to increase the risk of IPF [16, 17]. Genetic studies in familial and sporadic IPF patients have revealed an association between IPF susceptibility and telomerase-related genes [18], surfactant-associated genes [19, 20], mucin 5B gene, toll interacting protein (TOLLIP), and the signal peptide peptidase like 2C (SPPL2C) [21, 22]. Additionally, matrix metalloproteinase 1 (MMP1) and matrix metalloproteinase 7 (MMP7) are found in the peripheral blood of individuals with IPF and are highly overexpressed in lung fibrosis and reflect disease progression [23]. The activation of multiple wound-healing pathways is also a characteristic of IPF progression. Necrosis and/or apoptosis of alveolar epithelial cells is a consistent finding in patients with lung fibrosis [24–26]. Macrophages are involved in the wound-healing response in the lung and subsequent IPF development. The involvement of macrophages in IPF is another aspect of lung disease that needs evaluation to understand the role played by these inflammatory cells in the development of IPF.

### Macrophages in IPF

Although there is a continuum of macrophage polarization beyond the simplified, discrete, in vitro-based classification system, pulmonary macrophages can also be broadly classified into classically activated macrophages (M1 macrophages) and alternatively activated macrophages (M2 macrophages) depending on their functional phenotypes and biological activities [27]. M1 macrophages are induced by lipopolysaccharide (LPS), IFN-γ, and granulocyte–macrophage colony-stimulating factor (GM-CSF) and produce mediators aimed at eliminating foreign materials and debris. The transcription factor interferon regulatory factor 5 (IRF-5) promotes the M1 phenotype during early inflammatory stages to protect against intracellular pathogens by inducing nitric oxide synthase (iNOS) and proinflammatory cytokines such as IL-1b, IL-12b, IL-23, and TNF [28–30]. However, sustained inflammatory responses can trigger fibrotic responses in the lung. By contrast, M2 macrophages, which are induced by IL-4, IL-13, TGF-b, and IL-10 [31], are known to release mediators that downregulate the inflammatory response and promote the resolution of injury and tissue repair. M1 and M2 macrophages have distinct roles in the pathogenesis of pulmonary fibrosis due to their different cytokine expression profiles [10]. Usually, after alveolar epithelial injury, M1 macrophages heal the wound while M2 macrophages are responsible for completing the healing processes or inflammatory responses in the lung [10]. The reaction to unrelenting lung injury is alteration of wound healing process, which ultimately can result in IPF. Lung fibrosis progression has not been successfully halted by previous therapies due to a lack of knowledge of the exact mechanisms by which the balance between M1/M2 macrophage phenotype can be manipulated. Hence, future studies need to focus on the interactions between macrophages and fibroblasts.

## Macrophages derived from pluripotent stem cells

### Methods to derive macrophages from monocytes and TRMs

Several advancements have led to the development of different methods to derive generic macrophages, but there are very few methods available for the functional analysis of human M0s. The earliest method for macrophage generation involved direct isolation of tissue resident macrophages (TRMs) from relevant tissues [32–35]; however, insufficient yield and poor cell quality are major limitations of this method [36]. The use of immortalized cell lines from hemato-oncological patients such as THP-1 or U937 cells is a cheap and robust way to derive resting macrophages (M0s). However, the genetics of these malignant cells limits the application of this approach [37, 38]. Another method widely used for the generation of M0s exploits CD14 + monocytes derived from peripheral blood mononuclear cells. Monocytes are induced by growth factors or cytokines (mainly M-CSF) to generate M0s [39, 40]. An advantage of this method is the ready availability of human peripheral blood samples and the high quantity of monocyte-derived macrophages (MDMs) obtained from individual donors. However, MDMs cannot be used to model TRMs and are unable to proliferate and be maintained under culture conditions for long periods of time [4, 7, 41–44].

### Methods to derive macrophages from PSCs

Macrophages have also been derived from pluripotent stem cells (PSCs) such as embryonic stem cells (ESCs) and induced human pluripotent stem cells (iPSCs). Most of the methods used follow the common principle of stepwise differentiation of PSCs into M0s through the formation of mesoderm, hemogenic endothelium (HE), hematopoietic progenitors, and monocytic cells, mimicking embryonic hematopoiesis [45–48]. Generation of M0s from PSCs has several advantages over previously established human M0 models.

**Fig. 1:**
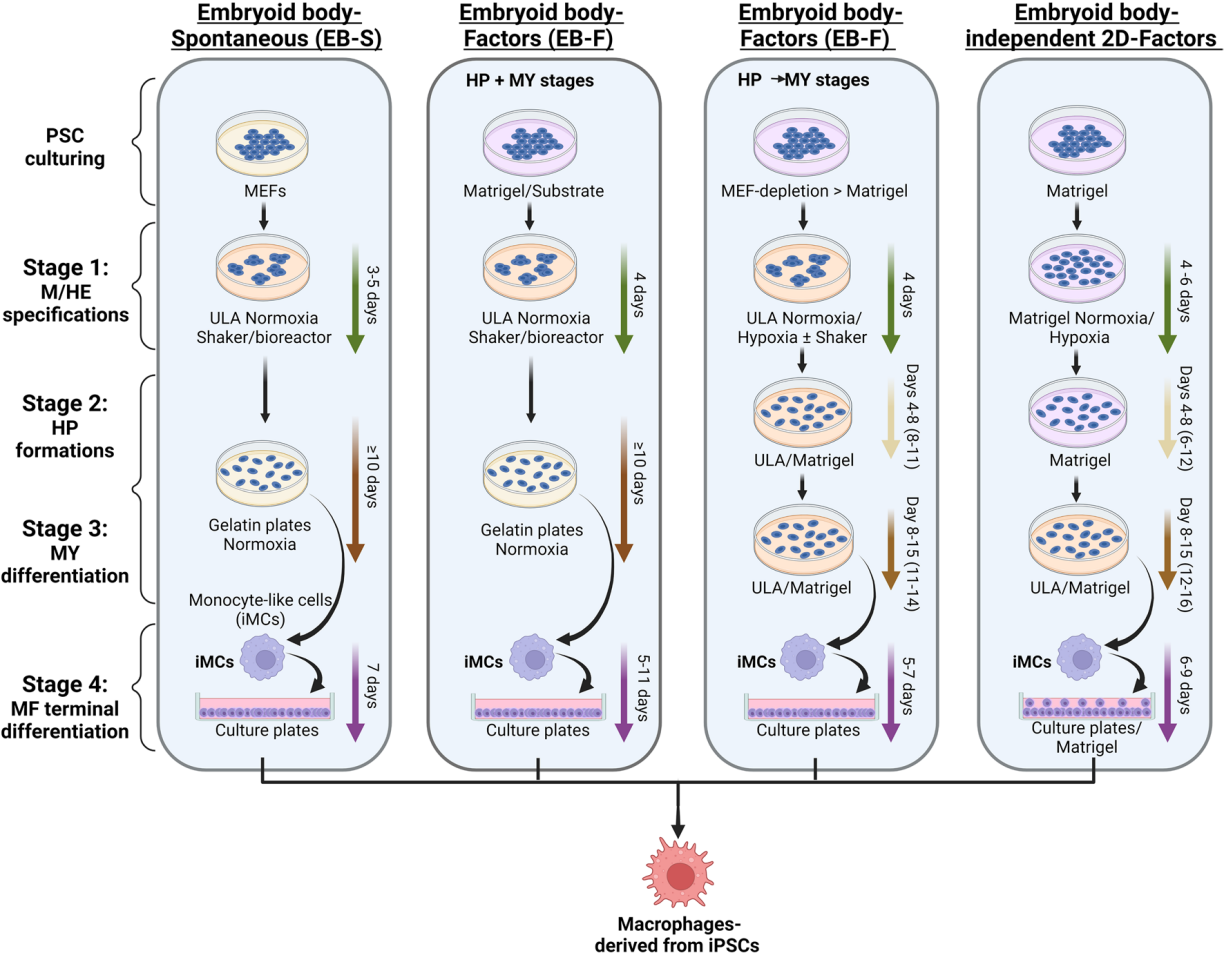
Schematic representation for the generation of iMphs from PSCs using different protocols. In all the protocols, the differentiation of PSCs into iMphs goes through four main stages as described. *Embryoid Body-Spontaneous (EB-S)* Step-1, PSCs culturing- PSCs are cultured and expanded on MEFs. Stage 1, M/HE stage- The mesoderm or hemogenic endothelium (M/HE) is induced through the EB formation in ultra-low adhesive (ULA) culture dishes. Stage 2 and Stage 3, HP and MY stages, the EBs formed in stage 1 are transferred to matrix-coated plates and cultured in medium supplemented with IL-3 and M-CSF under normoxia conditions. The floating monocyte-like cells (iMCs) are collected and transferred to new culture plates for their terminal differentiation. Stage 4, MF terminal differentiation- The cells collected from stage 3 are cultured using RPMI medium supplemented with M-CSF for their terminal differentiation into macrophages-derived from iPSCs (iMphs). *Embryoid Body-Factors (EB-F) HP* + *MY*: Step-1, PSCs culturing- PSCs are cultured and expanded on matrix-coated culture plates. Stage 1, M/HE stage- The mesoderm/HE specification in EBs is directed by externally supplied factors under normoxia condition. Stage 2 and Stage 3, HP and MY stages, the EBs from stage 1 are transferred to new plates and cultured in medium supplemented with IL-3 and M-CSF. Similar to EB-S protocol, the floating cells are collected and transferred to new culture plates for their terminal differentiation. Stage 4, MF terminal differentiation- The cells collected from stage 3 are cultured using RPMI medium supplemented with M-CSF for their terminal differentiation into iMphs. *Embryoid Body-Factors (EB-F) HP → MY* Step-1, PSCs culturing- PSCs are depleted from the MEFs before subjecting to differentiation. Stage 1, M/HE stage- The mesoderm/HE specification in EBs is directed by combination of exogenous factors under normoxia or hypoxia conditions. Stage 2, HP stage- The EBs obtained from stage 1 are transferred to either ULA or matrix-coated plates and cultured in the presence of specific exogenous factors to induce HP stage. Stage 3, MY stage- In the MY stage, the composition of exogenous factors is modified for the generation of iMCs. Stage 4, Stage 4, MF terminal differentiation- The floating iMCs cells are collected from stage 3 and cultured with M-CSF for their terminal differentiation into iMphs. *Embryoid Body-independent 2D-Factors* Step-1, PSCs culturing- PSCs are cultured on Matrigel-coated plates. Stage 1, M/HE stage- The M/HE is induced by culturing cells in M/HE-specific factors on matrigel coated plates under normoxia or hypoxia condition. Stage 2, HP stage- The HP specification is achieved in the presence of HP-specific factors. Stage 3, MY stage- For MY differentiation, the cells are either transferred to ULA plates or matrigel-coated plates and cultured under a set of exogenous factors for further differentiation into iMCs. Stage 4, MF terminal differentiation-The floating iMCs cells are collected from stage 3 and cultured with M-CSF for their terminal differentiation into iMphs

This approach can be used to generate ideal TRM models compared with MDMs due to the easy accessibility and scalability of PSCs. Several protocols have been established to differentiate PSCs into M0s (iMphs), all of which comprise four major stages: (1) mesoderm commitment and HE specification (M/HEstage); (2) endothelial-to-hematopoietic transition and the generation of hematopoietic progenitors (HP stage); (3) myeloid specification and the formation of monocyte-like cells (MY stage); and (4) terminal differentiation of iMphs (MF stage). There are several protocols that differ in the methods used to culture and differentiate PSCs that are outlined below. In 2D-OP9 protocols, PSCs are co-cultured with stromal cells to differentiate into M0s. S17, OP9, C166, UG26, and AM20.1B4 are different types of stromal cells that secrete factors that promote the proliferation of hematopoietic cells [49]. OP9 was successfully used for iMph differentiation due to its ability to prevent early monocyte/M0s bias and differentiation into different types of hematopoietic lineages. Given that OP9 cells are derived from the bone marrow cells of osteopetrosis mice, the use of xenogeneic cells limits its therapeutic applications. Stromal cell-independent protocols were developed later and there are three different protocols based on the induction of mesoderm commitment and HE specification (M/HE). Embryoid body (EB)–based three-dimensional (3D) spontaneous protocols (EB-S) generate EBs by culturing PSCs under low-adherent conditions while M/HE differentiation is achieved without the addition of exogenous factors [50–53]. Further, HP and MY differentiation are induced simultaneously by treatment with a combination of cytokines (IL-3 and M-CSF). EB-based 3D factor-assisted protocols (EB-F) involve exposure of EBs to several exogenous factors such as BMP4, SCF, and VEGF to achieve M/HE specification [48, 51]. HP and MY differentiation are induced simultaneously with IL-3 and M-CSF. Some EB-F protocols sequentially differentiate the HP stage into the MY stage. HP differentiation is achieved by treating cells with hematopoietic factors without M-CSF followed by conditional MY differentiation using hematopoietic factors along with M-CSF. EB-independent two-dimensional (2D) factor-assisted protocols (2D-F) rely on culturing PSCs on matrix-coated plates (matrigel) with several external factors (BMP-4, VEGF, SCF, FGF2, CHIR99021, and activin A) for M/HE specification [54–57], followed by sequential differentiation into HP and MY stages. Finally, all protocols involve terminal differentiation from monocyte-like cells to iMphs by cultivating cells in the presence of M-CSF. The schematic representation for the protocols and the detailed information of the exogenous factors supplemented along with the culture medium are discussed in Fig. 1 and Table 1, respectively.

**Table 1:** Culture medium and exogenous factors supplied during iPSCs differentiation into iMphs

Protocol	Stage	Exogenous differentiation factors	Medium
EB-S	PSC expansion	FGF2	KO-DMEM
			DMEM/F12
			AdvDMEM
	Stage 1	NA	KO-DMEM
			DMEM/F12
			AdvDMEM
	Stage 2 and 3	IL3 + M-CSF	X-VIVO 15
	Stage 4	M-CSF	X-VIVO 15 RPMI
EB-F HP + MY	PSC expansion	FGF2	mTeSR1
			DMEM/F12
	Stage 1	BMP4/VEGF/SCF	mTeSR1
			DMEM/F12
	Stage 2 and 3	IL3 + M-CSF	X-VIVO 15
	Stage 4	M-CSF	X-VIVO 15 + RPMI
EB-F HP → MY	PSC expansion	FGF2	DMEM/F12
			AdvDMEM
	Stage 1	BMP4/VEGF/SCF/ FGF2/Flt3L/TPO/CHIR/ActA	StemPro-34
			mTeSR1
			(KO-DMEM)
	Stage 2	VEGF/SCF/ FGF2/Flt3L/TPO/IL3/M-CSF	StemPro-34
	Stage 3	VEGF/SCF/ FGF2/Flt3L/IL3/IL6M-CSF/GM-CS	StemPro-34
			RPMI
	Stage 4	M-CSF	RPMI
2D-F	PSC expansion	NA	mTeSR1
			E8
			TeSR-E8
	Stage 1	BMP4/VEGF/ FGF2/ CHIR/ActA/S B/SCF	StemPro-34
			mTeSR1
			E8
			IMDM/F12
	Stage 2	VEGF/ FGF2/SCF/TPO/Flt3 L/IL3/IL6	StemPro-34
			IMDM/F12
			(DMEM/F12)
	Stage 3	FGF2/SCF/Flt3 L/IL3/IL6/ MCSF	StemPro-34
			IMDM/F12
			(DMEM/F12)
	Stage 4	M-CSF	IMDM/F12
			RPMI

## PSC-derived macrophages as disease models

Several studies have identified the potential of using PSC-derived macrophages in disease modelling of several diseases. iMph-based disease models are generated mainly through two major approaches. In the first approach, iMphs are generated using patient-derived iPSCs. This method has been used to model Gaucher disease, familial Mediterranean fever, Alzheimer disease, Tangier disease, chronic granulomatous disease (CGD), and Parkinson’s disease [58–62]. In the second approach, iMphs are generated using healthy donors-derived iPSCs mutated to carry the disease-associated mutations. This method has been successfully used to model CGD [63] and very-early onset bowel disease (VEOBD) [64, 65].

PSC-derived macrophages have also been used as a standard model to study macrophage-pathogen interactions and determine the role of human genetics in disease outcomes. Two closely related mosquito-transmitted flaviviruses (Zika virus and Dengue virus) have been studied using iMphs [66]. PSC-derived macrophages are highly relevant to study infectious agents that require macrophages as a source of persistence and replication, such as *Chlamydia* [67], *Salmonella* [68], and HIV [69]. These studies suggest that M0s models can therefore be utilized to determine the impact of infectious agents on disease pathogenesis and persistence.

Recent emergence of three-dimensional (3D) model systems, such as organoids and spheroids, and hydrogel systems have advanced our understanding of the tissue microenvironment better than two-dimensional (2D) models. The intact 3D structure allows replication of cell–cell and cell–matrix interactions to study regenerative medicine, disease modelling, and drug development in a variety of diseases. In this review, we outline the current 3D models to elucidate cellular and molecular cues in IPF and drug discovery.

### 3D models of IPF and drug discovery

Despite significant research, the development of effective therapies for IPF faces challenges due to the lack of in vitro models to mimic disease pathophysiology. Lung 3D cultures including precision cut lung slices (PCLS), hydrogels, and lung organoids have emerged as valuable tools for drug discovery and testing in a variety of pulmonary diseases including IPF[70–73]. PCLS maintain the native lung environment and is a relevant in vitro model to study lung fibrosis and drug testing in a diseased tissue condition [74]. Hydrogels are water-swollen cross-linked networks of polymers and offer another in vitro model to study IPF. Hydrogels can be customized to model normal or diseased microenvironments by altering biomaterials and crosslinking mechanisms [73]. The generation of lung organoids using hPSCs is a recent method to study the development of fibrosis and to screen novel drugs. hPSC-derived lung organoid models retain patient gene mutations and allow an improved understanding of molecular mechanisms of fibrosis. Strikoudis et al. demonstrate the importance of hPSC-derived lung organoids to model fibrotic lung disease wherein they identified the therapeutic potential of interleukin-11 (IL-11) in lung fibrosis [75]. Korogi et al. also provide insight into the potential role of hiPSC-derived lung organoids in disease modelling [76]. Cystic fibrosis patient-specific iPSC-derived lung epithelial cells have been used as an in vitro model to test novel small-molecule compounds called cystic fibrosis correctors [77]. The therapeutic value of NP-011 in assessing the anti-fibrotic potential of milk fat globule-EGF factor 8 (MFG-E8) protein has been demonstrated by hPSC-derived multicellular alveolar organoids containing functional alveolar epithelial and mesenchymal cells as an in vitro model for pulmonary fibrosis [70]. hPSC-based alveolar organoids also served as a relevant in vitro model to evaluate the toxic effect of particulate matter (PM2.5) on fetal alveolar development and acute respiratory syndrome coronavirus clade 2 (SARS-CoV-2) susceptibility [78].

A major drawback of these organoids is lack of immune cell components, such as macrophages, which limits the recapitulation of *in vivo* cellular physiology to model IPF and design subsequent drug screening approaches. Multicellular alveolar organoids with macrophages (Mac-AOs) exhibit phenotypic and functional resemblance to human macrophages and can demonstrate critical pulmonary fibrosis pathological features such as inflammation, collagen accumulation.

## Macrophage-derived extracellular vesicles

**Fig. 2:**
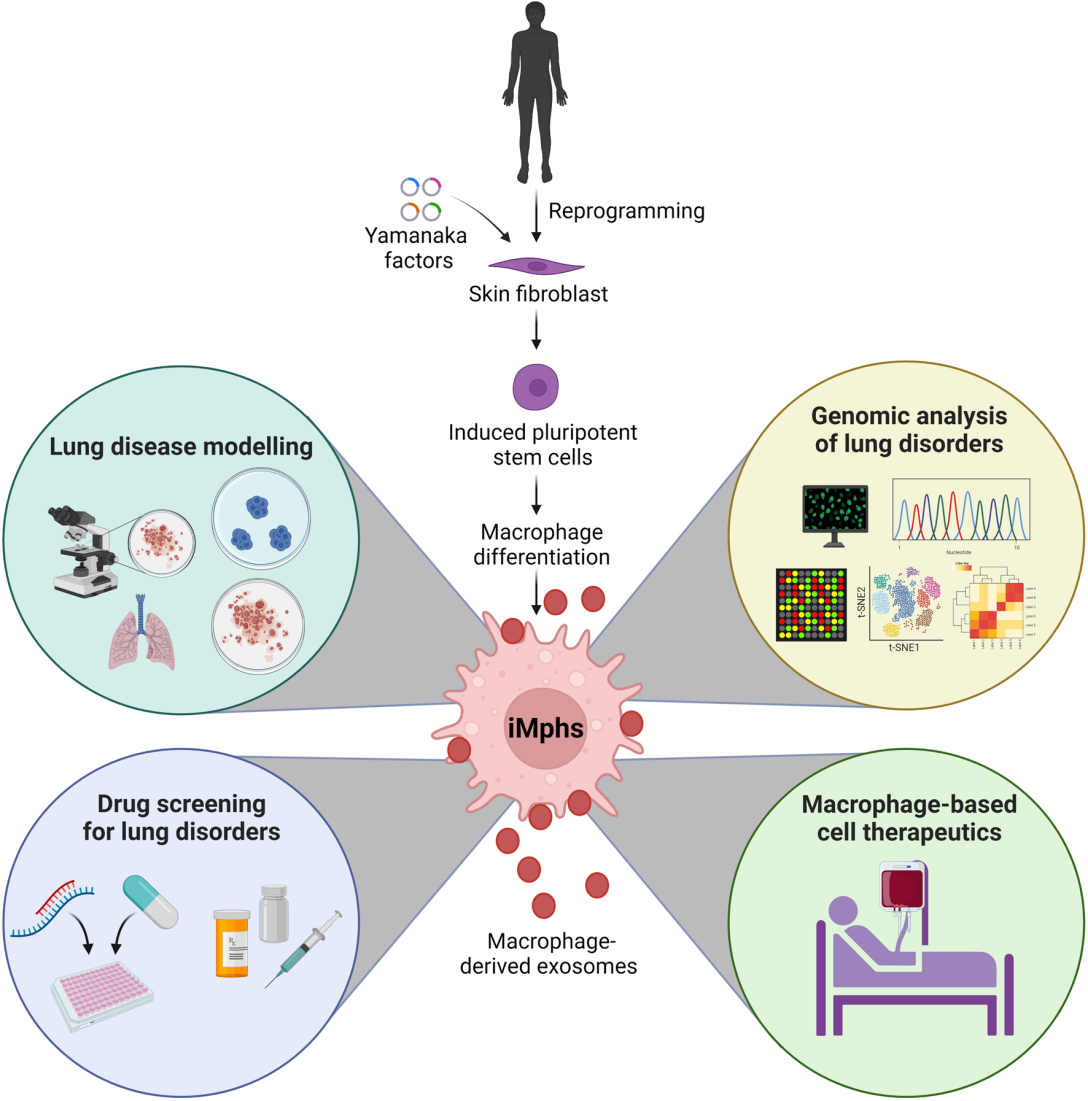
A representative scheme for macrophage generation from iPSCs and their current and prospective applications in genomic analysis of lung disorders, lung disease modelling, drug screening specific to lung disorders and macrophage-based cell therapeutics to lung disorders

Macrophages are blood immune cells that reside in all tissues and constitute the first line of defense against invading pathogens. Any abnormality in macrophage responses may result in uncontrolled inflammation and immune disorders, which are implicated in many diseases including renal inflammation and fibrosis [79]. Activated macrophages communicate with target cells via direct cell-to-cell contact and/or release of cytokines and extracellular vesicles (EVs) to exert their immunomodulatory functions. EVs are membrane-enclosed vesicles that can be classified as large oncosomes (LOs, ~ 1–10 μm), apoptotic bodies (ABs, ~ 1–5 μm), microvesicles (MVs, ~ 200–1000 nm), exosomes (Exos, ~ 30–200 nm), or exomeres (< 50 nm) on the basis of their size [80]. EVs exhibit similar properties to their parent cells and function as vital carriers to transfer cargo such as proteins, nucleic acids, and metabolites from parent cells to recipient cells. The role of macrophage-derived EVs, especially exosomes, has been widely investigated in different diseases to determine the roles played by EVs in disease progression. In this review, we primarily focus on the roles of macrophage-derived exosomes in disease and therapy (Fig. 2).

Exosomes are cell-secreted, nanosized, bi-lipid vesicles continuously secreted from various types of cells including alveolar epithelial cells, fibroblasts, and inflammatory cells [81]. Exosomes contribute to biological processes by transporting various bioactive molecules, such as nucleic acids (including miRNAs), proteins, and lipids [82–87]. Exosomes regulate various inflammatory and angiogenic pathways by transferring miRNAs from a donor to a recipient cell [83, 88–90]. Exosomes are considered to promote the polarization of macrophages [91, 92]. A large proportion of microvesicles in the blood are derived from macrophage-derived exosomes [93]. Exosomal-enclosed miRNAs have been shown to play crucial roles in inflammation, tissue repair, and fibrogenesis [94]. MicroRNAs are small non-coding single-stranded RNAs containing 18–25 nucleotides that are widely distributed in various organisms from viruses to humans [95] To date, more than 1000 human miRNAs have been found. These molecules act as regulators of gene expression by inhibiting protein translation, and play key roles in signal transduction, tissue and organ development, and other biological processes [96, 97]. Previous studies have demonstrated that exosomes play a pivotal role in various pulmonary diseases such as IPF, chronic obstructive pulmonary disease (COPD), and asthma [98–100].

### COPD

COPD is a chronic inflammatory lung disease caused by significant exposure to noxious particles or gases and is characterized by obstructive airflow resulting in breathing abnormalities. The symptoms of COPD are further exacerbated by several environmental factors such as smoking. Bronchial epithelial cells (BECs), the primary cells in contact with external stimuli such as cigarette smoke, play a critical role in airway homeostasis and are considered to be major EVs producer in the lung. Elevated levels of exosomes have been found to correlate with C-reactive protein (CRP), soluble tumor necrosis factor receptor-1 (sTNFR1), and interleukin (IL)-6 levels; all these molecules are plasma biomarkers of systemic inflammation with a potential pathophysiological role in COPD [101]. Exosomes secreted in COPD patients contain miRNAs (miR-210) that promote inflammation and alter the gene expression of target cells. Moreover, miRNAs from plasma-derived vesicles can serve as biomarkers in smokers and COPD patients [102].

### Asthma

Asthma is a common lung disease that affects both children and adults and that causes recurrent episodes of wheezing, breathlessness, and chest tightness [103]. Cells such as bronchial epithelial cells, dendritic cells, eosinophils, mast cells, and T-cells release cytokines in this disease [104–106]. Eosinophil-derived exosomes activate structural lung cells and contribute to the pathogenies of asthma [107]. Levels of serum exosomes containing miRNA-125b have been found to be higher in patients with asthma and may serve as a marker of asthma severity [108]. Studies in mice have revealed a protective role for M2 macrophage-derived exosomes. Under asthmatic conditions induced by ovalbumin, miR-370 levels were found to be reduced in M2 macrophage-derived exosomes and were found to be correlated with improved OVA-induced lung fibrosis and inflammatory responses [91].

### IPF

IPF is a chronic, progressive, and idiopathic interstitial pneumonia characterized by the replacement of healthy tissue by altered extracellular matrix and an impaired alveolar structure. Therapy for IPF focuses mainly on prolonging the life expectancy of patients by slowing down disease progression [109, 110]. For this reason, new pharmacological treatments and biomarkers to ensure better outcomes and diagnose patients early are needed. A recent study investigated the quantity of microRNAs in serum extracellular vesicles, including exosomes, of mice with bleomycin-induced lung fibrosis, and reported significant up-regulation of serum EV miR-21e5p in both the acute and chronic fibrotic phases. Furthermore, as miR-21e5p promotes TGF-b signaling, which is a key signaling pathway in IPF, miR-21e5p was suggested to be a potential biomarker of IPF [111]. EVs were also found to promote the proliferation and activation of fibroblasts in lungs and participate in the pathogenesis of IPF by mediating WNT5A signaling [112]. In another study, two types of miRNAs were found to be associated with IPF: (1) sputum macrophages were found to contain elevated levels of exosomal miR-142-3p in IPF while (2) macrophage-derived exosomes exerted a protective effect against pulmonary fibrosis progression via the delivery of antifibrotic miR-142-3p [83].

## Therapeutic potential of exosomes in chronic respiratory diseases

The therapeutic potential of exosomes extends to many diseases, including those of the lung, liver, kidney, and heart. Exosomes have been widely studied by several pharmaceutical companies to create products with therapeutic applications. MSC-derived exosomes developed by Aegle Therapeutics are under phase 1/2 clinical trials for dermatological disorders (NCT04173650). Another company, Carmine Therapeutics, derived EVs from RBCs to develop next-generation gene therapies to overcome the limitations of existing viral-based therapies such as low transgene capacity, immunogenicity, and other manufacturing challenges.

Exosome-based therapy is currently also being pursued in lung diseases such as IPF, COPD, acute lung injury (ALI), acute respiratory distress syndrome (ARDS), and bronchopulmonary dysplasia (BPD). MSC-derived exosomes have been used to treat ARDS [113] and protect against cigarette smoke-induced damage [114]. Intranasal delivery of exosomes derived from human amnion epithelial cells (hAECs) targets inflammatory and regenerative cascades of IPF to reverse lung fibrosis and enhance endogenous lung repair [115]. Similarly, inhalation of lung spheroid cell-secretomes (LSC-Sec) and exosomes (LSC-Exo) has been shown to be beneficial in treating lung fibrosis by reversing alveolar damage and decreasing myofibroblast proliferation and collagen accumulation [116]. Endothelial progenitor cell (EPC)-derived exosomes containing miR-126 attenuated LPS-induced ALI/ARDS and restored pulmonary integrity in a rat model of lung injury [117]. A mouse model study of hypoxia-induced pulmonary hypertension revealed that the cyto-protective role of MSCs in the lung is mediated by exosomes [118]. Exosomes have also been used to treat neonatal lung diseases. Treatment of a neonatal mouse model of hyperoxia-induced BPD with MSC-exosomes significantly improved lung morphology and associated pulmonary fibrosis [119]. Blood plasma-derived exosomes for early diagnosis of lung cancer (NCT04529915) and exosome-based identification of malignant and benign pulmonary nodules (NCT04182893) are currently being investigated in clinical trials. However, more research is needed to explore the therapeutic potential and niche of exosomes.

## Conclusions

Macrophages are critical players in maintaining homeostasis and immune responses to the external environment. Different types of macrophages secrete various pro-inflammatory and anti-inflammatory signals and affect several processes ranging from immune protection to wound healing [120–127]. The pro-inflammatory and anti-inflammatory functions of macrophages depend on the cellular microenvironment, which enables fine-tuning of the transcriptomic and functional response according to homeostatic needs. Any dysregulation in this balance can lead to inflammation, cancer initiation, cardiovascular disorders, and development of fibrosis. Thus, macrophages are potential therapeutic targets in various disorders; however, the lack of efficient models to replicate macrophages has limited their therapeutic value.

The groundbreaking discovery by Takahashi and Yamanaka of stem cells has led to their use to model disease progression, test drugs, and design individual-specific treatments. PSC-derived macrophages have become a highly attractive source for cell and gene therapy. The iMph approach has been successful used to correct genetic mutations and improve phagocyte functions in CGD and VEOBD [64, 65]. The development of novel therapeutic targets for diseases involving macrophages such as pulmonary alveolar proteinosis and liver fibrosis further expands the therapeutic applications of PSC-derived macrophages. Pulmonary transplantation of gene-edited host macrophages into a murine model of hereditary pulmonary alveolar proteinosis had beneficial therapeutic effects [128]. Transplantation of human iPSC-derived macrophages upon intrapulmonary development into AM-like cells resulted in a striking reduction of alveolar protein and surfactant D deposition and attenuated the hereditary pulmonary alveolar proteinosis (herPAP) phenotype [129]. TALEN-mediated integration of the corrected gene granulocyte–macrophage colony-stimulating factor receptor alpha-chain (CD116) into patient-specific iPSCs resulted in functionally compatible macrophages [130]. Intratracheal injection of human iMphs in an acute *P. aeruginosa* infection model enhanced pulmonary immunity [50]. Therapeutic application of macrophages significantly reduced the amount of hepatic fibrosis in a model of liver injury [131]. Further, the identification of an active compound against *M. tuberculosis* using iMphs to screen a 3,716-compound library expanded the therapeutic uses for PSC-derived macrophages as drug testing models [132]. Together, these studies suggest that PSC-derived macrophages are promising biomaterials for the treatment of several diseases (Fig. 2).

EVs derived from macrophages have become widely accepted as disease biomarkers and therapeutic tools. For example, the presence of immune molecules on the surface of EVs [133] and elimination of macrophage-associated risks such as cytokine release syndrome [134] have highlighted the potential of macrophage-derived EVs in therapeutics. Future research should focus on establishing efficient protocols to generate economically feasible, high-yield, clinically-applicable generic macrophages. Elucidating the specific roles of lung macrophages in fibrotic lung disorders would facilitate the development of effective macrophage-based therapeutics.

## Data Availability

Not applicable.

## Abbreviations

PF: Pulmonary fibrosis
hPSC: Human pluripotent stem cells
RBCs: Red blood cells
AMs: Alveolar macrophages
IMs: Interstitial macrophages
TOLLIP: Toll interacting protein
MMP: Matrix metalloproteinase
LPS: Lipopolysaccharide
GM-CSF: Granulocyte-macrophage colony-stimulating factor
IRF-5: Interferon regulatory factor 5
TRMs: Tissue resident macrophages
M0s: Resting macrophages
MDMs: Monocyte-derived macrophages
EB: Embryoid body
VEOBD: Very-early onset bowel disease
PCLS: Precision cut lung slices
MFG-E8: Milk fat globule-EGF factor 8
EVs: Extracellular vesicles
COPD: Chronic obstructive pulmonary disease
BECs: Bronchial epithelial cells
CRP: C-reactive protein
sTNFR1: Soluble tumor necrosis factor receptor-1
ALI: Acute lung injury
ARDS: Acute respiratory distress syndrome
BPD: Bronchopulmonary dysplasia
hAECs: Human amnion epithelial cells
herPAP: Hereditary pulmonary alveolar proteinosis
